# Design and Construction of “Synthetic Species”

**DOI:** 10.1371/journal.pone.0039054

**Published:** 2012-07-25

**Authors:** Eduardo Moreno

**Affiliations:** Institute of Cell Biology, University of Bern, Bern, Switzerland; The University of Queensland, St. Lucia, Australia

## Abstract

Synthetic biology is an area of biological research that combines science and engineering. Here, I merge the principles of synthetic biology and regulatory evolution to create a new species with a minimal set of known elements. Using preexisting transgenes and recessive mutations of *Drosophila melanogaster*, a transgenic population arises with small eyes and a different venation pattern that fulfils the criteria of a new species according to Mayr’s Biological Species Concept. The population described here is the first transgenic organism that cannot hybridize with the original *wild type* population but remains fertile when crossed with other identical transgenic animals. I therefore propose the term “synthetic species” to distinguish it from “natural species”, not only because it has been created by genetic manipulation, but also because it may never be able to survive outside the laboratory environment. The use of genetic engineering to design artificial species barriers could help us understand natural speciation and may have practical applications. For instance, the transition from transgenic organisms towards synthetic species could constitute a safety mechanism to avoid the hybridization of genetically modified animals with *wild type* populations, preserving biodiversity.

## Introduction

It has been argued that reconstructing a system is the ultimate way of understanding it [1], [2]. In order to further comprehend the origin of new species, and to explore possible applications in modern biotechnology, I engineered reproductive isolation between populations of *Drosophila melanogaster* by generating a synthetic species boundary.

The use of *Drosophila* is justified because this model organism is leading the fields of regulatory evolution and speciation [3], [11]. For example, previous work in several species of *Drosophila* produced fundamental contributions regarding the genetics of speciation [7]–[10]. In addition, key studies of evolution in *Drosophila* have shown that novelty arises more readily from the recruitment of existing elements into new regulatory networks than from the development of completely new components [3]–[6].

However, speciation has been given no attention as a tool for biotechnology and in the context of genetically engineered organisms. Previous artificial speciation experiments produced “incipient species” that were not fully isolated or whose speciation genes were unknown [12]–[16]. Reproductive isolation has been brought about in plants for many decades through polyploidization, which creates individuals that in crosses to parents give rise to sterile progeny [13], and, more recently, in yeast [14]. For the animal kingdom, a parthenogenetic species of lizard was also generated [15], but, again, its speciation genetics are not understood, does not involve transgenesis nor synthetic design and its components can therefore not be reliably and predictably manipulated.

Unlike previous artificial speciation events, the synthetic species boundary described here is a genetic circuit based on the combination of 5 well known preexisting elements leading to reproductive isolation. Building components so well understood that can be reliably and predictably manipulated is one of the goals of synthetic biology. In this case it allows opening and closing speciation gates when desired.

## Results

**Figure 1:**
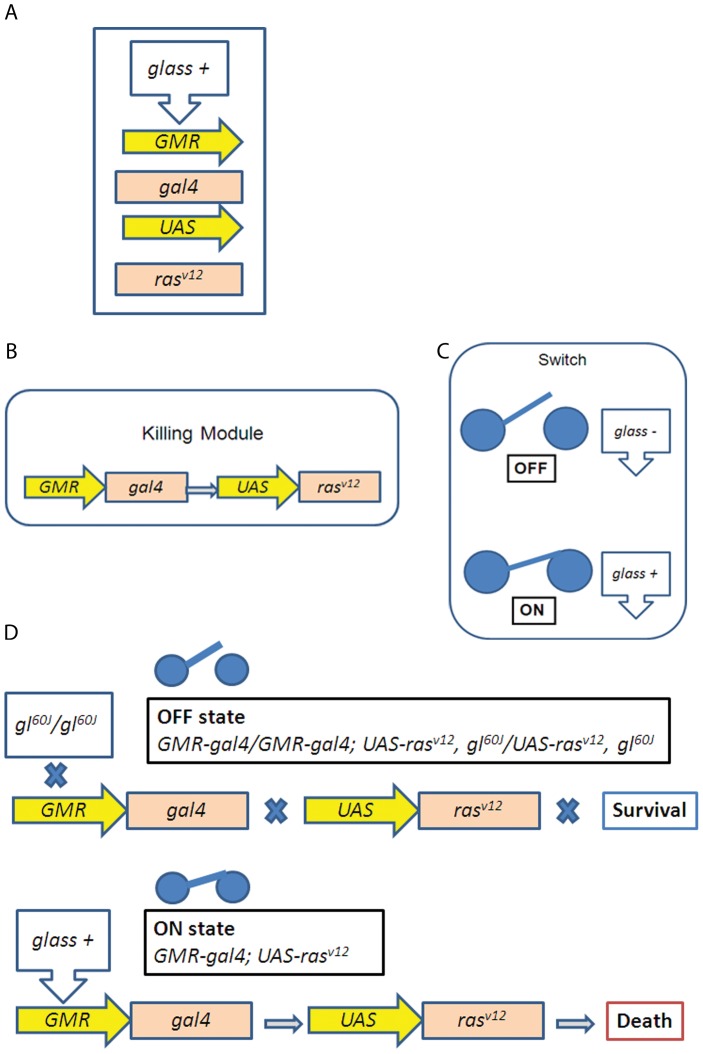
Design of a genetic circuit with selected components that form a synthetic species barrier. (A) The 5 genetic elements used: transcription factor *glass*, enhancer *GMR*, transcription factor *gal4*, enhancer *UAS* and a constitutively activated form of *ras*. (B,C) Arrangement of the genetic elements in two modules, a killing module (B) composed by two independent transgenes, *GMR-gal4* and *UAS-ras^v12^*,and a switch that depending on the presence or absence of the transcription factor *glass* can switch the killing module ON and OFF (C). (D) In the absence of Glass, activation of the killing module is not possible and the flies survive. However, in the presence of Glass, expression of the constitutively active form of *ras* kills the animal.

Regulatory evolution acts by using available preexisting genetic elements to generate novelty. Likewise, the synthetic genotype created here is achieved by implementing known elements, however their selection and specific arrangement establishes a previously unknown synthetic species barrier (Fig. 1).

The first element consist of null mutations in the *glass (gl)* gene [19], [20]. The *glass* product is a transcription factor of 604 amino acids with five zinc-fingers. Mutations in *gl* specifically abolish photoreceptor cells resulting in blind, but viable flies [19], [20]. The *gl^60J^* allele is a spontaneous mutant caused by the insertion of 30 kb of unknown DNA into the *gl* locus and it is believed to be a null allele [19], [20]. Other alleles (*gl^3^* and *gl^BS1^*) have also been used for this study.

The second element is formed by the Glass Multimer Reporter (GMR) [20], [21], a heterologous promoter construct containing five tandem copies of a 27-bp *glass*-binding site normally present in the regulatory region of *ninaE*, the major *rhodopsin* gene in *Drosophila*. The GMR promoter can therefore drive *glass*-dependent expression in the photoreceptor cells of *Drosophila* eyes.

The yeast protein GAL4 as a third building block can activate transcription in *Drosophila* from promoters that bear GAL4 binding sites [22], [23]. In addition, the GMR sequence has been previously subcloned in front of *gal4*, thus driving Gal4 expression under the control of Glass (*GMR-gal4*) [21].

Fourth, a tandem array of five GAL4 binding sites (5×UAS, for Upstream Activation Sequence) is employed where GAL4 binds with high affinity to induce the transcription of a downstream located gene.

The fifth element is a *ras^v12^* allele, a mutant form of the *Drosophila ras* gene [24], [25]. Conversion of the glycine residue at position 12 to valine constitutively activates the Ras protein. *ras^v12^* has been previously subcloned behind GAL4 binding sites (*UAS-ras^v12^*), which permits activation only within cells where GAL4 is expressed [24].

The inherent logic of the design relies on a “killing module” (Fig. 1b) and a regulator to switch it ON and OFF (Fig. 1c):

The killing module is formed by *GMR-gal4* and *UAS-ras^v12^* whose activation is controlled by the presence or absence of the gene *glass*. When *glass* gene function is unperturbed, transcription of *UAS-ras^v12^* driven by *GMR-gal4* consistently kills 100% of the flies at any temperature from 17°C until 29°C (606/606 lethality at 17°C, 558/558 lethality at 23°C, 330/330 at 25°C, 110/110 at 29°C, Table 1). Pupae arrest at mid pupation and due to abnormal tissue lysis the pupal case ends up almost empty.

**Table 1:** Crosses between *Drosophila synthetica* and *Drosophila melanogaster*.

Parental genotypes	F1 adult progeny	Number of dead pupae
♂ ***GMR-gal4/GMR-gal4***♀ ***UAS-ras^v12^/UAS-ras^v12^***	0 (no survivors at any temperature from17°C to 29°C)	606/606 lethality at 17°C 558/558 lethality at 23°C 330/330 lethality at 25°C 110/110 lethality at 29°C
♂*** GMR-gal4/GMR-gal4; UAS-ras^v12^, gl^60J^/UAS-ras^v12^, gl^60J^***♀ ***GMR-gal4/GMR-gal4; UAS-ras^v12^, gl^60J^/UAS-ras^v12^, gl^60J^***	>1000 at 17°C >1000 at 25°C	0 at 17°C 20/100 at 25°C
♂ ***GMR-gal4/GMR-gal4; UAS-ras^v12^, gl^60J^/UAS-ras^v12^, gl^60J^***♀ ***Oregon R***	0 (no survivors)	293/293 lethality at 17°C
♂*** Oregon R***♀*** GMR-gal4/GMR-gal4; UAS-ras^v12^, gl^60J^/UAS-ras^v12^, gl^60J^***	0 (no survivors)	50/50 lethality at 17°C
♂*** &*** ♀ ***w; GMR-gal4/GMR-gal4; UAS-ras^v12^, gl^60J^/*** ***UAS-ras^v12^, gl^60J^ (genotype 1)***♂ ***& ***♀ ***w/w (genotype 2)***	>1000 of genotype 1>1000 of genotype 2 0hybrids (red normally sized eyes)	>1000 at 17°C
♂ ***GMR-gal4/GMR-gal4; UAS-ras^v12^, gl^60J^/UAS-ras^v12^, gl^60J^***♀ ***tub-gal80/tub-gal80***	71 at 25°C	0 at 25°C
♂ ***GMR-gal4/GMR-gal4; UAS-ras^v12^, gl^60J^/UAS-ras^v12^, gl^60J^***♀ ***gl^60J^/gl^60J^***	87 at 25°C	0 at 25°C
♂*** &*** ♀ ***w; GMR-gal4/GMR-gal4; UAS-rasv12, gl60J/*** ***UAS-rasv12, gl60J (genotype 1)***♂ ***&*** ♀ ***y,w/y,w (genotype 2)***	99 of genotype 1 165 of genotype 2 0hybrids (red normally sized eyes)	140 at 17°C
♂ ***& ***♀ ***w; GMR-gal4/GMR-gal4; UAS-rasv12, gl60J/*** ***UAS-rasv12, gl60J (genotype 1)***♂ ***&*** ♀ ***y,w,f/y,w,f (genotype 2)***	30 of genotype 1 11 of genotype 2 0hybrids (red normally sized eyes)	24 at 17°C

More than 20 other *UAS* transgenes were tested, including *UAS-caudal* [26], *UAS-flower^LoseA^* [27] or *UAS-eiger* [27], but *UAS-ras^v12^* was the only one that resulted in 100% lethality when driven by the presence of *glass* and the *GMR-gal4* transgene at all temperatures (from 17°C to 29°C) (Table 1).

**Figure 2:**
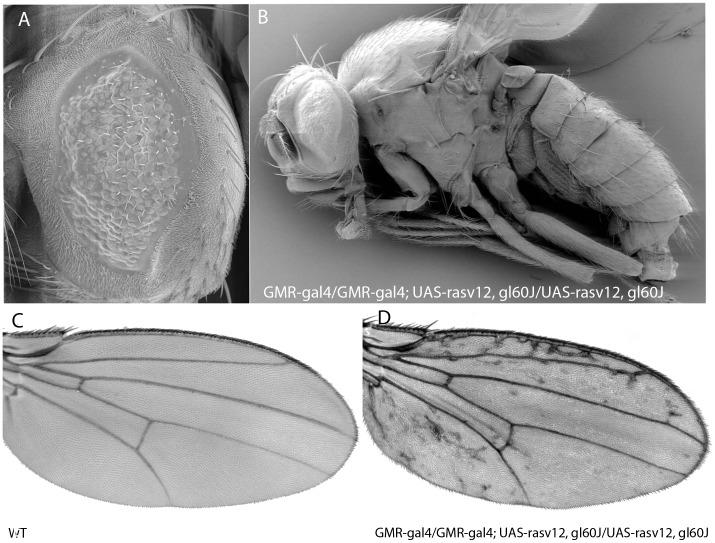
Morphological traits of *Drosophila synthetica*. (A–B) Scanning electronic microscopy (SEM) images of *Drosophila synthetica* flies. Eye is small due to lack of *glass*. (C–D) Wings of *Drosophila synthetica* show extraveins in the lateral regions of the wing (D) compared to the *Drosophila melanogaster* wing (C).

In the synthetic genotype *GMR-gal4/GMR-gal4; UAS-ras^v12^, gl^60J^/UAS-ras^v12^, gl^60J^* (a *glass^60J^* mutant background, where no Glass protein is present) Ras^v12^ cannot be produced (OFF state, Fig. 1d). Surprisingly however, in addition to the small eye phenotype (Fig. 2a,b), those flies showed a different wing morphology, with lateral extra veins (Fig 2d, compare with the wt wing pattern shown in 2c). Other alleles (*gl^3^* and *gl^BS1^*) were also tested and yielded the same phenotype. Most likely the heat shock promoter (*hsp70*) of the *GMR-gal4* construct is leaky, leading to very low activation of *UAS-ras^v12^* and consequently to the phenotype [28].

**Figure 3:**
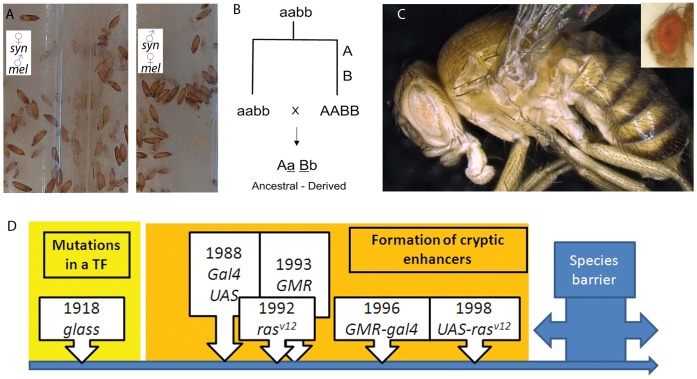
Creation of species boundaries by regulatory evolution. (A) Hybrids between *melanogaster* and *synthetica* arrest in pupae and do not develop further, even at 17C. The sex of the parents did not affect the outcome. Pupae shown in the pictures are more than one month old. (B) Scheme of a classical Dobzhansky-Muller mechanism for speciation, where all mutations occur in one of the populations (“derived”), and the hybrids between the “ancestral” (aabb) and “derived” (AABB) populations are lethal. (C) High definition and depth of field images of *Drosophila synthetica* after several generations of coexistence with *D. melanogaster*. Image obtained with a Keyence VHX-600 microscope. Eyes are pale in addition to small. A *D.melanogaster* eye is shown for comparison in the upper right corner. (D) General model for the creation of species boundaries based on the modification of transcription factors and the subsequent appearance of cryptic enhancers. This could be a mechanism to create synthetic species and prevent hybridization of transgenic animals with natural populations. The case of *Drosophila synthetica* is shown. Years correspond to the first appearance of the mutation or transgene in a *Drosophila* laboratory.

When hybrids between *Drosophila melanogaster* and the synthetic genotype are produced, the “killing module” *GMR-gal4; UAS-ras^v12^* is triggered by the presence of the *glass* gene (Fig. 1d, Table 1, Fig. 3a). This genetic network, while still allowing normal reproduction among flies with the synthetic genotype, completely isolates *GMR-gal4/GMR-gal4; UAS-ras^v12^, gl^60J^/UAS-ras^v12^, gl^60J^* flies from normal *D. melanogaster* due to hybrid early pupal lethality (Fig. 3a, Table 1). Unlike with the other known and naturally occurring speciation mutations [8], the sex of the parents did not influence the lethality of the hybrids in this case (Table 1, Fig. 3a). Experiments were performed at 17°C because flies of the synthetic genotype grew better and because due to the temperature sensitiveness of the Gal4 it is likely to be the temperature at which the killing module may be less effective. Despite this, the killing module was 100% effective even at 17°C (Table 1, Fig 3a). In the initial population mutations in *yellow (y^1^)*, which results in mild pigmentation, existed as a polymorphism in some individuals.

It is often difficult to delineate “species boundaries” since they may carry identical mutations and are related to one another through common ancestors. However, most biologists agree on a very stringent definition for species, the Ernst Mayr’s Biological Species Concept, according to which species consist of populations of organisms that can reproduce with one another, but are reproductively isolated from other such groups [7] (Fig. 3b). This definition leads to a focus on the barriers to reproduction between species [8]–[14]. Such barriers represented one of the main problems for Darwin who wrote: “How can we account for species, when crossed, being sterile (…), whereas, when varieties are crossed, their fertility is unimpaired?” [11]. Because the postzygotically isolated population generated here conforms to the most stringent definition of species [8]–[14], it will subsequently be called *Drosophila synthetica*.

To further prove that the synthetic genetic network allowed zero gene flow with *D. melanogaster,* co-cultures of both populations were performed for 13 generations (using *D.melanogaster white (w)* mutants with white eyes) and not a single hybrid was recovered (Fig. 3c, Table 1). Hybrids would have been easily recognizable by normally sized red eyes, because they would carry a normal copy of *gl* and two *w+* copies from the transgenes (Table 1), but *D. synthetica* behaved like a stable species, did not interbreed and maintained its characteristic eyes (Fig. 3c). Identical results were obtained when crossing *D. synthetica* with other *melanogaster* strains, including *y,w* flies and *y,w,f* flies (Table 1). In all cases *synthetica* and *melanogaster* did not interbreed (Table 1).

Assembling synthetic species boundaries can have practical applications. For example, the use of recombinant DNA technology to alter organisms for a specific purpose has raised controversy [18] and is a growing problem due to the increasing number of transgenic organisms approved by regulatory agencies [16]–[18]. A new framework where safety mechanisms are genetically designed along with desired modification could help to gain public support for a technology with the potential to satisfy future medical and nutritional needs [16]–[18]. *D. synthetica* is the first transgenic organism that cannot reproduce with the original *wildtype* population. I therefore propose that synthetic species barriers may serve to compartmentalize dangers and protect natural species from interbreeding with emergent transgenic forms, therefore preserving natural biodiversity (Table 1).

Moreover, once a genetic network is identified, as is the case for the “*ras-glass*” synthetic boundary described here, opening or closing of the barrier can be controlled at will. In case the interbreeding of populations appears beneficial, targeted strategies can be implemented to reverse hybridization barriers. To test this experimentally, the GAL4-inhibitor GAL80 was expressed from a *tubulin* promoter (*tub-gal80*) [29] in *D. melanogaster* in order to remove hybrid lethality and traverse the species barrier. Males of *D. synthetica* hybridized successfully with *tub-gal80 D. melanogaster* females and produced viable hybrids (Table 1), as predicted because Gal80 can block the “killing module”.

## Discussion

The postzygotically isolated population generated here in the genus *Drosophila* conforms to the most stringent definition of species [8]–[14], as well as to the principles of synthetic biology [1], [2], and it has been consequently named *Drosophila synthetica*. I propose the term “synthetic species” to distinguish it from “natural species”, not only because it has been created in the laboratory, but also because it may never be able to survive in the wild, unlike “natural species”. For example, because the flies created are blind and only survive at lower temperatures, they have potential fitness deficits and it could be argued that the changes could not be arrived at in concert because the “fitness valley” will not be traversed in the wild. However, blindness is a common adaptation in caves suggesting that fitness deficits are difficult to predict and depend on environmental conditions [7], [11], [27].

Interestingly, the generation of *Drosophila synthetica* matches the Dobzhansky-Muller theoretical model for postzygotic incompatibilities during naturally occurring speciation [7], [8], according to which an ancestral population splits into two independent populations that then accumulate mutations (Fig. 3b). Subsequent genetic interactions between those mutations cause hybrid incompatibilities. In particular, it conforms to a derived-ancestral incompatibility (Fig. 3b), in which all substitutions occur in the derived population.

I therefore propose that modifications in transcription factors and appearance of cryptic enhancers upstream of potentially lethal gene products can constitute a normal Dobzhansky-Muller mechanism for speciation (Fig. 3d). The appearance of those cryptic enhancers could be driven by the accumulation of point mutations in regulatory regions (Fig. 3d), in a manner similar to what has been described recently [30], but those enhancers will only be recognised by the ancestral transcription factor which is now missing (or modified) in the derived population (Fig. 3d). When hybridization between the derived and ancestral populations occurs, the genes with cryptic enhancers will be activated by the ancestral transcription factor, causing hybrid lethality and reproductive isolation (Figs. 1,2, Table 1). This could constitute a general mechanism through which regulatory evolution creates species boundaries (Fig. 3d) and may help to define concrete target genes mediating speciation.

One of the predictions of classical evolutionary theory is that organisms that connect two species must exist as part of the gradual divergence process [11]. Because we fully know the mutations forming a reproduction barrier between *melanogaster* and *synthetica*, it is feasible to dissect the process and move backwards, showing how populations of intermediate mutants can indeed interbreed with populations at either side of the evolutionary path towards postzygotic isolation (i.e., *glass* mutants can hybridize with both species (Table 1) and hence connect *melanogaster* with *synthetica,* as if they were a “missing link”) (Fig. 3).

The results shown here provide proof of principle for the transition from “transgenic animals” to “synthetic species”, as defined above, and should spur the debate for its use as a failsafe mechanism in biotechnology. Modifying the binding affinities of one transcription factor and the enhancers it recognises, could be used to engineer reproductive isolation in other living animals, not only in *Drosophila*. Moreover, the ability to open and close speciation gates when desired reflects one of the goals of synthetic biology –to build components that can be reliably and predictably manipulated–, and preserves flexibility while gaining control over the spread of genetically modified organisms.

One potential caveat could be that this barrier is not irreversible since it can be overturned quite simply if a spontaneous mutation was to arise in any of the components. However, if we think in terms of engineering (or synthetic biology), having fail safe mechanisms in a machine makes it safer; despite they may stop working. The solution is to add more fail safe devices. Identically, adding more synthetic speciation barriers will increase safety. Other transcription factors and enhancers could be easily used to create those extra barriers, because the concept goes beyond any particular element. Importantly, modification of the binding properties, instead of complete elimination of the transcription factor, could also be implemented, reducing the constraint of not finding enough non-essential transcription factors to build several barriers.

In summary, the synthetic species boundary described here can be reliably and predictably manipulated, allowing opening and closing speciation gates when desired, and isolates for the first time a transgenic animal from the original wild-type population.

## Methods

High definition and depth of field photographs were obtained with a Keyence VHX-600 microscope. Flies were frozen at −20°C overnight before imaging. For SEM, adults were fixed in 2.5% glutaraldehyde in PBS overnight at 4°C, post-fixed in 1% osmium for 2 h at 4°C, washed, dehydrated in ethanol and with Hexamethyldisilazane until evaporation of the solvent. Samples were coated with 30 nm of gold and observed with a 440 Leica microscope under 20 kV tension.

The fly stocks used were obtained from the Bloomington Stock Center except where indicated. The following stocks were used: *GMR-gal4, UAS-ras^v12^, glass^60J^, UAS-Dpp, UAS-wg-HA*, *UAS-egr, UAS-brk (*G.Campbell*), UAS-hep^CA^, UAS-fwe^Lose-A^* and *UAS*-*fwe^Lose-B^*, *UAS-hid* (H. Steller), *tub-GAL80.*

For the balancing of the different transgenes the following stocks were used:

*ywhs-FLP;If/CyO; MKRS/TM6b.*

*w^1118^; Pas^SC1^ gl^3^/TM6B, gl^BS1^ Tb^1^.*

*w^1118^; If/CyO; MKRS/TM6B, gl^BS1^ Tb^1^.*

*C(1)DX,y^1^,f^1^,hs-hid.*

### Nomenclatural Acts

The electronic version of this document does not represent a published work according to the International Code of Zoological Nomenclature (ICZN), and hence the nomenclatural acts contained in the electronic version are not available under that Code from the electronic edition. Therefore, a separate edition of this document was produced by a method that assures numerous identical and durable copies, and those copies were simultaneously obtainable (from the publication date noted on the first page of this article) for the purpose of providing a public and permanent scientific record, in accordance with Article 8.1 of the Code. The separate print-only edition is available on request from PLoS by sending a request to PLoS ONE, Public Library of Science, 1160 Battery Street, Suite 100, San Francisco, CA 94111, USA along with a check for $10 (to cover printing and postage) payable to “Public Library of Science”. The online version of this work is archived and available from the following digital repositories: PubMed Central, LOCKSS.
